# Muscle Thickness and Echogenicity Measured by Ultrasound Could Detect Local Sarcopenia and Malnutrition in Older Patients Hospitalized for Hip Fracture

**DOI:** 10.3390/nu13072401

**Published:** 2021-07-13

**Authors:** Alejandro Sanz-Paris, Mikel González-Fernandez, Luis Enrique Hueso-Del Río, Eduardo Ferrer-Lahuerta, Alejandra Monge-Vazquez, Francisco Losfablos-Callau, Teresa Sanclemente-Hernández, Alejandro Sanz-Arque, Jose Miguel Arbones-Mainar

**Affiliations:** 1 Department of Endocrinology and Nutrition, Miguel Servet Hospital, 50009 Zaragoza, Spain; gonzalezmikel@hotmail.es (M.G.-F.); flosfablos@gmail.com (F.L.-C.); 2 Centro de Investigación Biomédica en Red Fisiopatología Obesidad y Nutricion (CIBERObn), Instituto Salud Carlos III, 28029 Madrid, Spain; jmarbones.iacs@aragon.es; 3 Adipocyte and FatBiologyLaboratory (AdipoFat), Translational Research Unit, University Hospital Miguel Servet, 50009 Zaragoza, Spain; 4 Department of Internal Medicine, Miguel Servet Hospital, 50009 Zaragoza, Spain; lehuerio@hotmail.com; 5 Department of Radiology and Ultrasound, Miguel Servet Hospital, 50009 Zaragoza, Spain; edeli2107@gmail.com; 6 Department of Traumatology and Orthopedics, Miguel Servet Hospital, 50009 Zaragoza, Spain; amongev@salud.aragon.es; 7 Nutrition Department, Zaragoza University, 5009 Zaragoza, Spain; tsanclem@unizar.es; 8 Centro de Salud Santa Ana, 31500 Tudela, Spain; jandrosanzarque@hotmail.com; 9 Instituto Aragonés de Ciencias de la Salud (IACS), Instituto de Investigación Sanitaria, Aragón (IIS-Aragón), 50009 Zaragoza, Spain

**Keywords:** ultrasound, hip fracture, malnutrition, regional sarcopenia

## Abstract

Background: The aim of this work was to assess whether the muscle thickness and echogenicity were associated with dysphagia, malnutrition, sarcopenia, and functional capacity in acute hospital admission for a hip fracture. Methods: Observational study that assessed nutritional status by Global Leadership Initiative on Malnutrition, risk of dysphagia and sarcopenia by European Working Group on Sarcopenia in Older People and Barthel functional index. We measured muscle thickness and echogenicity of masseter, bicipital, and quadriceps rectus femoris (RF) and vastus intermedius (VI) by ultrasound. Results: One hundred and one patients were included in the study (29.7% sarcopenia and 43.8% malnutrition). Logistic regression models adjusted for age, sex, and body mass index showed an inverse association of the masseter thickness with both sarcopenia (OR: 0.56) and malnutrition (OR: 0.38) and quadriceps with sarcopenia (OR: 0.74). In addition, patients at high risk of dysphagia had lower masseter thickness (*p*: 0.0001) while patients able to self-feeding had thicker biceps (*p*: 0.002) and individuals with mobility on level surfaces higher thickness of biceps (*p*: 0.008) and quadriceps (*p*: 0.04). Conclusion: Thickness of the masseter was associated with risk of dysphagia, biceps with the ability to self-feed, and that of the quadriceps RF-VI with mobility.

## 1. Introduction

Hip fracture is one of the most serious geriatric processes because of its high morbid-mortality and increase economic burden [1]. It is becoming more prevalent due to the increase in elderly populations [2]. The number of hip fractures increased by 36.9% and the cumulative mortality after 1 year of a hip fracture occurrence ranges between 20 and 40% from 2008 to 2012 [3].

Older people with hip fracture have a higher risk for being malnourished. In a recent review of Malafarina et al. the prevalence of malnutrition varied from 18.7% using the MNA questionnaire to 45.7% using the BMI, albumin, or weight loss. Regardless of the diagnostic method, malnutrition was associated with a worse clinical progression and increased mortality in elderly patients with a hip fracture [4]. Muscle mass loss is one of the most critical consequences of malnutrition, often associated with a deficit of protein intake, which limits muscle maintenance and increases the risk of sarcopenia [5]. Lower muscle mass has also a negative impact on the clinical progression of other chronic diseases decreasing overall survival, functionality, and quality of life [6]. In a recent revision, Inoue et al. found that 7% to 26% of patients with hip fracture were undernourished while 11% to 76.4% had sarcopenia. However, none of the reviewed studies used GLIM criteria as diagnosis for malnutrition [7].

Body composition can be assessed by computed axial tomography and magnetic resonance imaging, although these techniques are not commonly used in normal clinical practice due to the radiation and cost respectively. Traditionally, bioimpedance analysis (BIA) and anthropometric measurements of arm and calf diameter have been used to determine muscle mass of patients with malnutrition [8].

Ultrasound measurement is being implemented for muscle assessment in patients of different profile, including for sarcopenia screening [8,9]. One of the hallmark findings in sarcopenia is a tissue remodeling in which healthy muscle is replaced by fat and fibrotic tissue. These changes are reflected in an increase in echogenicity from higher sound transitions in the muscle. For this work we investigated the main muscles associated with nutrition and sarcopenia. We explored the masseter muscle for its role in chewing and swallowing, the biceps for its flexor movement to bring the food to the mouth, and finally the quadriceps for its importance in deambulation. On the other hand, muscle function is as important as muscle mass in elderly patients with malnutrition [10]. Tests like manual pressure strength (hand grip) and the get up and walk test (gait speed) have an important role in sarcopenia definition [10]. We hypothesize that ultrasound measurement can provide not only muscle mass by measuring its thickness, but also its functionality by assessing its echogenicity [11].

Therefore, the objective of this work was to demonstrate the suitability of the evaluation of muscle ultrasound for the diagnosis of regional sarcopenia associated with malnutrition, sarcopenia, and functional capacity in acute hospital admission for hip fracture. To this end, we used muscular ultrasound and the GLIM criteria for the diagnosis of malnutrition.

## 2. Materials and Methods

### 2.1. Study Design and Population

Cross-sectional observational study in patients admitted to a third level hospital for a hip fracture. Subjects were consecutively recruited during the first 24 h of hospital stay, from January to March 2020. Inclusion criteria were the absence of any other on-going acute process or terminal illness and a life expectancy greater than 6 months. Subjects receiving treatment with steroids, chemotherapy, hemodialysis or severe renal, liver or heart failure were excluded. Similarly, patients whose severe mental confusion or delirium was assessed with the Confusion Assessment Method [12], did not allow impedance or muscle ultrasound were excluded. Co-morbidities were determined by the Charlson index [13]. The protocol for this work was approved by our local ethical committee (Ethical Committee for Clinical Research of Aragon, CEICA ref # C.P.—C.I. PI19/487). All subjects and caregivers were informed of the study and signed their consent to participate.

### 2.2. Study Outcomes

#### 2.2.1. Nutritional and Anthropometric Assessment

In a first step, we screened malnutrition using the mini-nutritional assessment (MNA-SF) short form test [14]. Then, we diagnosed malnutrition according to the GLIM criteria [15], which require that at least one phenotypic and one etiologic criterion should be simultaneously present. For phenotypic GLIM criteria we assessed the following variations: (1) unintentional weight loss > 5% within the last 6 months; (2) body mass index (BMI) < 22 kg/m^2^; (3) reduction of muscle mass based on Appendicular Skeletal Muscle Index (ASMI). The ASMI cut off were < 7 kg/m^2^ for male or < 5.5 kg/m^2^ in females according to European Working Group on Sarcopenia in Older People [10]. We considered that all participants met the GLIM etiologic criteria for acute injury due to recent hospitalization for hip fracture. Their caregivers corroborated all information provided by the patients. We collected anthropometric parameters such as BMI and the circumferences of the arm and calf of the non-fractured side using a precision tape measure.

#### 2.2.2. Functional Assessment

Functional capacity prior to hip fracture was determined using the Barthel index in activities of daily living [16]. The individuals were graded in 10 activities that are added to give a score from 0 (totally dependent) to 100 (totally independent), applying the cut-off point 60.

Patients, or their caregivers if needed, completed the Spanish language validated version of the screening questionnaire EAT-10. Scores of 3 or higher were considering abnormal and suggestive of dysphagia [17]. Additional analyses were performed with scores 15 or higher because they are associated with aspiration and pneumonia risk [18].

#### 2.2.3. Sarcopenia Evaluation

The prevalence of sarcopenia was determined using the diagnostic criteria for age-related sarcopenia from the 2019 European Working Group on Sarcopenia in Older People (EWGSOP2) [10]. We considered sarcopenia as probable when low hand grip strength (HGS) was detected. A sarcopenia diagnosis was confirmed by BIA. Briefly, muscle strength was assessed by Jamar dynamometer (Jamar Hydraulic Hand-grip Dynamometer, model 5030J1, Duluth, MN, USA), using the best of three attempts with the dominant hand. Muscle mass was measured by BIA (Akern BIA 101 SMT device, Florence, Italy). The measurement protocol was performed according to the National Institutes of Health [19]. The subjects were in a supine position 5 min before the measurement, which was performed in a thermoneutral environment of 25 °C, with an empty bladder and stomach. Then we placed two electrodes on the foot of the non-fractured limb (in the theoretical line bisecting the malleolus and the ankle and at the level of the distal metatarsal) and other pair of electrodes on the homo-lateral hand (in the theoretical line bisecting the radius and the ulna, and other at the distal metatarsal level). Akern software provided Resistance (Rz) and Reactance (Xc) at 50 kHz. Phase angle was calculated directly from reactance and resistance (Phase angle = arc − tangent reactance/resistance × 180°/π).

Electrical parameters were converted to appendicular skeletal muscle mass/ height^2^ (ASMI) using the cross-validated Sergi equation with age, sex, weight, and height [20], based on older European populations. As recommend EWGSOP2 sarcopenia cut-off points for ASMI used was < 7.0 kg/m^2^ for men and < 5.5 kg/m^2^ for women and < 27 kg for men and < 16 kg for women for HGS [10].

#### 2.2.4. Muscle Ultrasound Exploration

**Figure 1:**
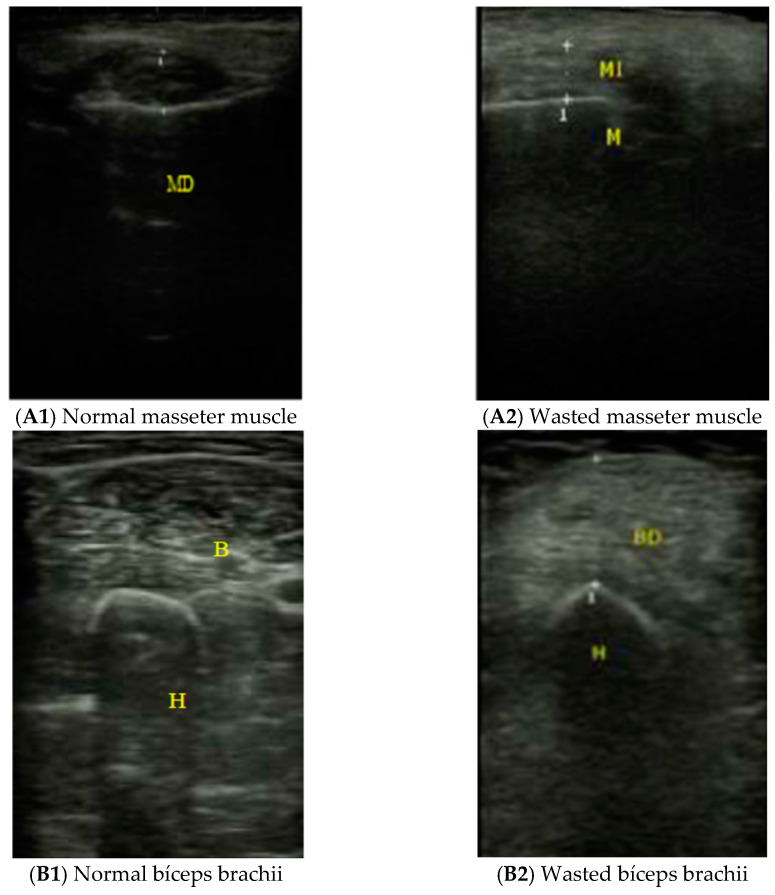

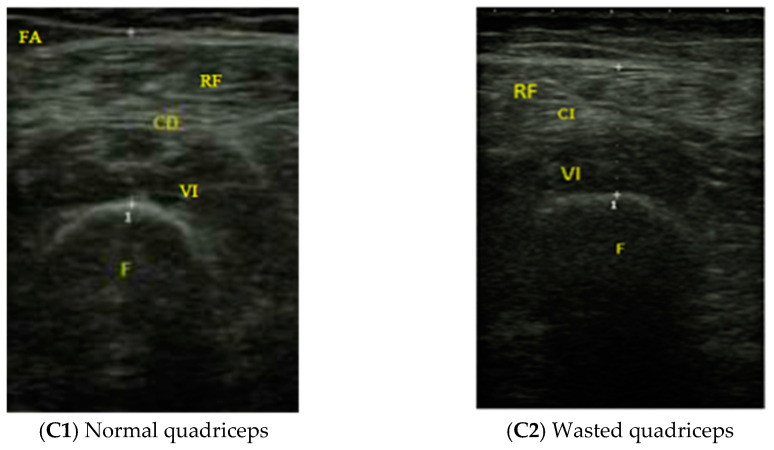
Ultrasound images. (**A**) Masseter muscle (A1 normal and A2 wasted). **A1** (normal masseter muscle), **A2** (wasted masseter muscle), (M: masseter muscle; MD: right jaw; MI left jaw); (**B**) Biceps brachii (B1 normal and B2 wasted). **B1** (normal bíceps brachii), **B2** (wasted bíceps brachii), (H: humerus; B: Biceps); (**C**) Quadriceps femoris (C1 normal C2 wasted). **C1**(normal quadriceps RF-VI), **C2** (wasted quadriceps RF-VI), (FA: subcutaneous celular tissue; RF: rectus femoris; VI: vastus intermedius; F: femoris CD: right quadriceps humerus; B: Biceps).

For the ultrasound determinations, an EDAN DUS 60 ultrasound scanner with a 7.5 MHz linear transducer (San Diego, CA, USA) was used. This technique was performed blindly as the operator did not know the results of the rest of the parameters evaluated in the study. The thickness of the masseter muscle was obtained in the mid-point between the tragus and the buccal commissure (Figure 1). We also measured the thickness of two out of four muscles constituting the quadriceps femoris: the rectus femoris and the vastus intermedius (quadriceps RF-VI) as indicated by Sabatino et al. [21] because it was difficult to separate one muscle from another in highly sarcopenic patients. The thickness and echogenicity of the quadriceps RF-VI were assessed in non-fractured limb, between the anterior superior iliac spine and the proximal end of the patella with the patient lying in supine position and the ultrasound probe placed perpendicularly with slight pressure [21]. Once the muscle tissue was identified, the distance was measured between the femoral cortex and the most superficial muscle fascia. The thickness of the brachial biceps was obtained at two-thirds of the way between the acromion and the antecubital crease with the transducer placed perpendicular and exerting minimum pressure [22]. We quantify the echogenicity using Image J (https://imagej.nih.gov/ij/; accessed on 6 April 2020).

### 2.3. Statistical Analyses

A descriptive analysis of the demographic and clinical characteristics of all the patients included in the study was carried out. To determine the normality of the variables a Kolmogorov-Smirnov goodness of fit test was performed. Of the quantitative variables that did not follow a Gaussian distribution of data the medians and interquartile range were shown, while frequencies and proportions were obtained for the qualitative variables.

For the comparison of means, a univariate analysis was performed by the Mann-Whitney U and Kruskal-Wallis tests according to the number of categories of analysis. For the comparison of qualitative variables, contingency tables were used with the chi-square test. We used the Spearman’s rank correlation coefficient (Rho) to check the statistical association between quantitative variables.

Logistic regression models were performed to determine the risk of sarcopenia based on the three muscles of interest and malnutrition diagnosis, as well as functional capacity. Models were adjusted for age, sex, and BMI. The results were expressed as odds ratios (OR) with 95% CI.

The data were analyzed with the statistics package SPSS 24 (Chicago, IL, USA). Statistical significance level was set at *p* < 0.05.

## 3. Results

### 3.1. Study Population

From 120 patients admitted for hip fracture from January to March 2020 in our hospital, 101 patients were included in the study (71 women) (Table 1). Nineteen patients were excluded for different reasons: not agreeing to participate (*n*: 3), agitation due to dementia (*n*: 5), and lower limb edema that made difficult to perform impedance (*n*: 4), decompensated congestive heart failure (*n*: 3), cirrhosis (*n*: 1), and advanced kidney failure (*n*: 3). The median (interquartile range) age was 86 (9) years; Barthel functional index 86 (24) and hospital stay 5 (3) days. Median Charlson morbidity index was 7 (3): 29.9% had some degree of kidney failure, 26.9% diabetes, 26.9% heart failure, 17.9% peripheral arterial disease, 16.4% dementia, 10.4% cerebrovascular disease, and 9% chronic obstructive pulmonary disease. The most frequently used drugs were cardiotonics, antiarrhythmics, anticoagulants, vasodilators, antiplatelets, oral hypoglycemics or insulin, and bronchodilators. Regarding the risk of dysphagia measured with EAT-10, 87 patients had no risk (< 3 points) while 14 patients were at risk (>3 points), of whom ten patients were at high risk (>15 points). There were no differences between sexes in any functional parameter from Barthel index.

**Figure 2:**
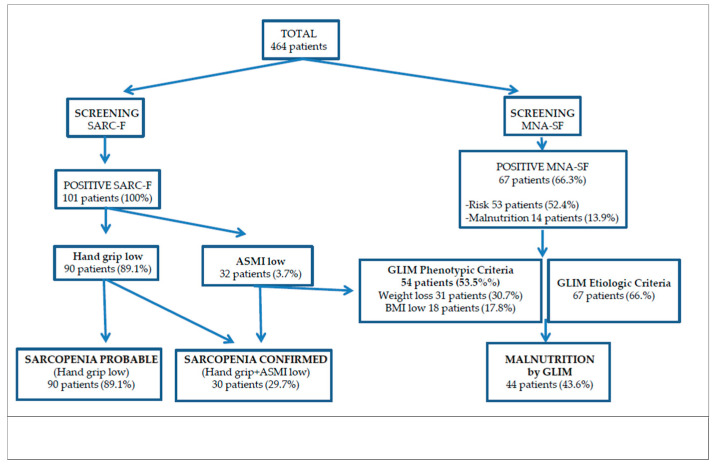
Flow chart for the diagnosis of sarcopenia and malnutrition.

Probable sarcopenia, defined by low hand grip strength, was present in 90 patients (89.1%) at admission but only 30 patients (29.7%) had confirmed sarcopenia by BIA. Regarding patients’ nutrition status, 66.3% were either malnourished (13.9%) or at risk of malnutrition (52.4%), according to the MNA-SF screening tool, and 43.6% had confirmed malnutrition by the GLIM criteria (Figure 2).

Compared to men, female showed more prevalence of malnutrition risk (*p*: 0.025) and a higher grade of echogenicity in the masseter muscle (*p*: 0.001). On the other hand, we found more prevalence of sarcopenia in men (*p*: 0.001). But we did not found sex differences in malnutrition prevalence by GLIM criteria or muscle thickness.

The BIA data are presented in detail in Table 1, with no sex differences in ASMI, Rz, Xc nor phase angle (PA).

### 3.2. Interrelations of Thickness and Echogenicity of the Three Muscle Groups Studied by Ultrasound

We observed a positive and statistically significant correlation between the thickness of the quadriceps RF-VI and bicipital muscles (Rho = 0.47, *p* = 0.001), while no significant correlation was found with the thickness masseter muscle. In regard of muscle echogenicity, masseter echogenicity was positive and statistically significantly correlated with biceps (Rho =0.5, *p* = 0.0001) and quadriceps RF-VI (Rho = 0.34, *p* = 0.01) echogenicity, as well as biceps with quadriceps RF-VI echogenicity (Rho = 0.68, *p* = 0.0001).

### 3.3. Relations of Thickness and Echogenicity of the Three Muscle Groups Studied by Ultrasound with Age and Anthropometric Assessments

Age correlated only positively with masseter echogenicity (Rho = 0.4, *p* = 0.003) and negatively with biceps thickness (Rho = −0.33, *p* = 0.01).

BMI correlated positively with thickness and negatively with echogenicity of the three muscles (Rho = 0.37 to 0.31 and −0.41 to −0.28, respectively). Arm circumference correlated negatively with echogenicity of the three muscles Rho = 0.23 to 0.21) and positively with thickness of biceps and quadriceps RF-VI (Rho = 0.4, *p* = 0.002 and Rho = 0.47, *p* = 0.0001, respectively). But calf circumference only correlated positively with biceps thickness (Rho = 0.4, *p* = 0.002).

### 3.4. Associations of Thickness and Echogenicity of the Three Muscle Groups Studied by Ultrasound with Nutritional Assessment

In regard of malnutrition screening, the thickness of the three muscle groups was greater in patients without risk of malnutrition according to MNA-SF, but only the thickness of the masseter muscle showed a significant association in the multivariate logistic regression analysis. Adjusted for age, sex, and BMI, we found that each 1 mm increase in masseter reduced the risk of malnutrition, according to MNA-SF, an ~70% (OR: 0.27, *p*: 0.001, 95% CI 0.13–0.56).

In malnutrition diagnosis, as well as in malnutrition screening, only the masseter thickness reached statistical significance in the multivariate logistic regression analysis. Adjusted for age, sex, and BMI, we found that each 1 mm increase in masseter reduced the risk of malnutrition by GLIM criteria in ~60% (OR: 0.39, *p*: 0.001, 95% CI 0.22–0.68) (Figure 2).

### 3.5. Associations of Thickness and Echogenicity of the Three Muscle Groups Studied by Ultrasound with Sarcopenia Diagnosis

**Figure 3:**
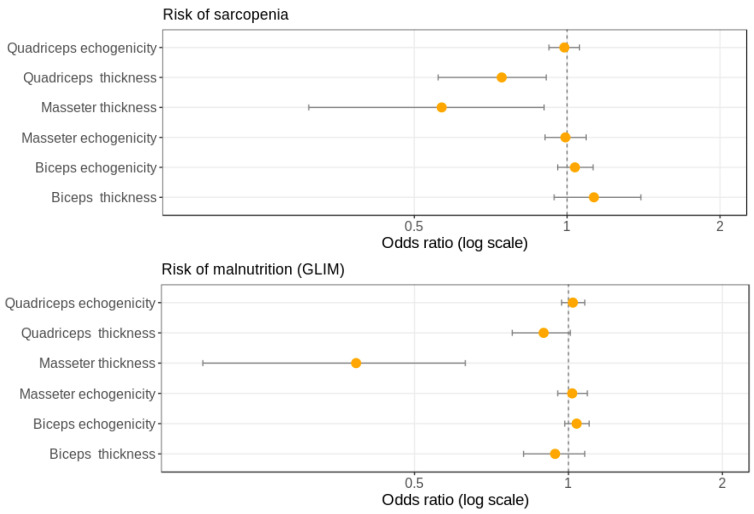
Forest plot of the multivariate adjusted model (logistic regression) of significant odd ratios for risk of sarcopenia and malnutrition with thickness and echogenicity of the three muscle groups studied by ultrasound.

ASMI correlated positively with the thickness of masseter, biceps, and quadriceps RF-VI muscles (Rho = 0.37, *p* = 0.005, Rho = 0.4, *p* = 0.003 and Rho = 0.55, *p* = 0.0001, respectively), but not with echogenicity. On the other hand, HGS correlated negatively with masseter and biceps echogenicity (Rho = 0.45, *p* = 0.001 and Rho = 0.43, *p* = 0.002, respectively), but no significant correlation was found between handgrip and any thickness muscle. Multivariate logistic regression analysis indicated that each 1 mm increase in masseter or quadriceps RF-VI muscles reduced the risk of sarcopenia by ~45% or 25% respectively (OR 0.56, *p*: 0.03, 95% CI 0.34–0.95) (OR 0.74, *p*: 0.01, 95% CI 0.58–0.94) (Figure 3).

### 3.6. Relations of Thickness and Echogenicity of the Three Muscle Groups Studied by Ultrasound with Functional Assessment

With respect to function and ultrasound results, we observed a good relationship between the thickness of each muscle group and the specific functional capacity. Thus, non-dysphagic individuals (86.1%) had thicker masseters compared to their dysphagic-risk counterparts (because EAT-1 > 3 points): 11.8 (2.5) vs. 8.4 (0.9) mm, *p*: 0.0001, and less echogenicity: 30.7 (11.4) vs. 40.2 (10.9), *p*: 0.02. In the subgroup with high risk of dysphagia (by EAT-10 >15 points), we observed similar results (masseter thickness *p*: 0.001 and echogenicity *p*: 0.026).

**Figure 4:**
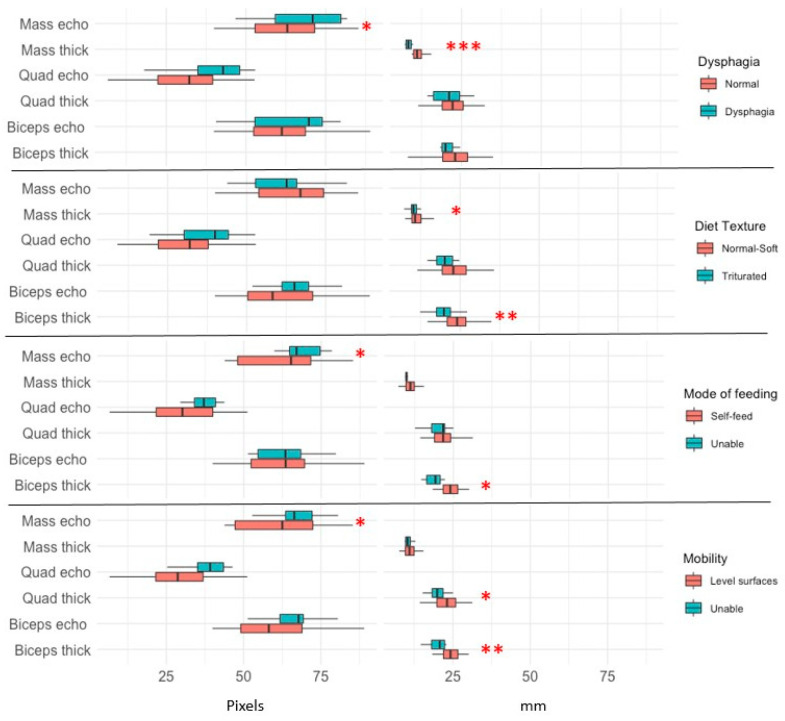
Comparison of muscle thickness and echogenicity of three muscles measured by ultrasound and functional assessment. Mass echo: masseter echogenicity, Mass thick: masseter thickness, Quad echo: quadriceps RF-VI echogenicity, Quad thick: quadriceps RF-VI thickness, Biceps echo: biceps brachii echogenicity, Biceps thick: biceps brachii thickness. * *p* < 0.05, ** *p* < 0.01, *** *p* < 0.001.

Furthermore, we found that patients with triturated diet showed lower masseter thickness (*p*: 0.02). The study of Barthel index factors showed that patients who could feed themselves had thicker biceps than those who could not (24.3 (4) vs. 18.6 (2.9) mm, *p*: 0.002). In regard of mobility, we observed higher thickness of biceps (*p*: 0.008) and quadriceps RF-VI (*p*: 0.04) in individuals with mobility on level surfaces compared to those unable to ambulate. In contrast, the echogenicity of the quadriceps RF-VI was higher in patients who could not climb stairs compared to those who had no difficulty (54.3 (12.1) vs. 67.9 (10.2), *p*: 0.003), without differences in muscle thickness (Figure 4).

## 4. Discussion

The main findings of this study were that in elderly patients hospitalized for a hip fracture the thickness and echogenicity data of the masseter, biceps, and quadriceps RF-VI muscles were related to each other and in different intensity with the nutritional status, diagnosis of sarcopenia, and their specific functional capacity. Ultrasonography is hence a novel approach to test the association of muscle thickness and echogenicity with function.

Most studies in elderly have described an association between muscle mass measured by ultrasound as a screening for sarcopenia [9,23], although we have not found studies in patients with recent hip fracture. In our study, we found that the thickness of the masseter muscle was related to dysphagia, biceps with the ability to eat independently and mobility, and quadriceps RF-VI with mobility on level surfaces and ability to climb stairs. All of this indicates that during the nutritional assessment, the ultrasonographic measure of muscle thickness would indicate the functional status of the localized area.

Sex, age and BMI are factors associated with muscle assessment, so that they are taken into account for the cut-off points in the diagnosis of sarcopenia and in the normality tables for any nutritional parameter [24]. In our study, age and BMI correlated negatively with thickness and positively with echogenicity of all muscle groups, so they were the factors chosen to adjust the multivariate logistic regression analysis.

It seems appropriate to use anthropometric measurements in a first approach to the muscle mass assessment because of the simplicity and reduced cost of these tests. Arm and calf circumferences are components of the MNA, which is the most validated nutritional assessment test in older adults [14]. In our study we observed an association of medium degree (Rho = 0.31 to 0.47), fundamentally between arm circumference and thickness (positive) and echogenicity (negative) of the biceps and RF-VI quadriceps. Some studies have reported a high correlation between anthropometric measures and muscular mass measured by dual X-ray absorptiometry [25]. In the same way, acceptable ultrasound correlations have been observed between the muscle mass of the biceps and arm circumference [26], and between the rectus femoris thickness and calf circumference [27]. However, in a recent study [28] the correlation between calf circumference and muscle thickness of the medial gastrocnemius was non-significant in those with excess weight. The authors suggest that ultrasound may offer greater precision in the quantification of skeletal muscle in the calf, since subcutaneous fat is excluded.

There are multiple studies about ultrasound measurement of muscle thickness as a test for sarcopenia assessment in elderly individuals [9,29] but the studies that relate it to nutritional assessment are very scarce. Our study used GLIM consensus as a nutritional assessment tool, finding a prevalence of malnutrition of 43.8%. We have only found one study using GLIM criteria in patients with hip fracture; Probert et al. [30] found a prevalence of malnutrition of 59% in 2008 and 37% in 2018 among Swedish patients with hip fracture. Our study observed correlations of medium-low magnitude for the result of the ultrasounds of the arm and limb muscle groups with the nutritional screening by MNA-SF (Rho = 0.26 to 0.36). This is in line with a previous study in institutionalized older adults describing a relationship between MNA-SF and the lower limb muscles (medial gastrocnemius and tibialis anterior) measured with ultrasound [27]. About nutritional assessment by GLIM criteria, the thickness of the three muscle groups was lower in malnourished patients, but only the masseter thickness reached statistical significance in the multivariate logistic regression analysis. Regarding the chewing muscles, we found two studies in Japanese older adults, which observed a good relationship between nutritional status and ultrasound findings of the temporal [31] and masseter muscles [32].

The prevalence of sarcopenia in older people admitted in acute units because of hip fractures varies between 17% and 37% [33,34]. In our study, the prevalence of sarcopenia was found to be 29.3%. Ultrasound as a screening tool in sarcopenia has been supported by a number of studies in critically ill patients and elders [35], but those on hip fracture are very rare. We have only found one study with 40 older adults admitted to the geriatric rehabilitation wards after hospitalization for a hip fracture [36]. The authors observed a decrease in the size of the rectus femoris in all patients, while the prevalence of total low muscle mass by BIA only affected 35%. Nevertheless, ultrasound should not be directly compared with the measures of ASMI by BIA with muscle strength. Ultrasound data could offer additional value about “tissue quality.” Hence, it could complement, but not replace, BIA measures. Additionally, ultrasound data must be validated by MRI using a study design that includes cross-validation.

Our results show that ASMI correlated with the three muscle thickness in medium grade (*p*: 0.37 to 0.55) while HGS correlated negatively with the three muscle echogenicity in medium grade (*p*: 0.26 to 0.45). These data could indicate that the thickness of the muscle measured by ultrasound could be related to the amount of muscle while echogenicity would be related to its function. Yamada et al. in 347 Japanese community-dwelling older men found that quadriceps muscle thickness was a better indicator of muscle mass, whereas its echo intensity was a more robust indicator of muscle function [37].

When we studied the association of the sarcopenia risk, we observed that it reduced as quadriceps RF-VI thickness increases (OR 0.74, *p*: 0.01). This suggests that large muscles such as the RF-VI quadriceps could be better indicators than small ones for representing total muscle mass. Furthermore, the measurement of a large muscle group such as the quadriceps RF-VI was related to other muscle mass (biceps and masseter), but small muscles such as the masseters or biceps were not related to each other. However, there is still a need for studies with the aim of establishing normality parameters by age and sex, and at the same time determine the specific importance of the muscle selected on the quality of the ultrasound intervention [29]. Thus, our results showed a relation between quadriceps RF-VI thickness and sarcopenia. It coincides with other works where the size of the peripheral muscles is a good indicator of overall muscle mass or they point to greater muscle loss by sarcopenia in the lower limbs [38].

Nagano et al. observed that dysphagia developed after hip fracture surgery could be sarcopenic dysphagia [39]. In our study, the multivariate logistic regression analysis showed that the thickness of the masseters is a protective factor against sarcopenia (OR 0.56, *p*: 0.03) and malnutrition by GLIM criteria (OR 0.38, *p*: 0.001). In elderly care home residents, Gaszynska et al. observed that masseter muscle thickness was positively associated with hand grip strength, body cell mass index, BMI, and activities of daily living [40]. In the same line, Umeki et al. [41] showed a significant association between masseter thickness and ASMI in community-dwelling elders. Murakami et al. [42] also observed an association between the decline in masticatory function and the incidence of sarcopenia. It is worth noting that masseter thickness is associated with both malnutrition and sarcopenia revealing itself as an important link between those conditions. In our study we observed a significant reduction of masseter thickness in patients with low and high risk of dysphagia (*p*: 0.0001 and *p*: 0.001) and triturated diet (*p*: 0.02), which are known factors of malnutrition [43]. In a previous study in 469 institutionalized elderly people, we showed a strong association between masseter thickness and dysphagia [44].

We also studied the relationship of the thickness of the other muscles and their respective functions. In our study, 1 mm increase in the thickness of biceps reduced half the odds of not being able to self-feed (OR: 0.5, *p*: 0.02) and by 30% the odds of having reduced mobility (OR: 0.7, *p*: 0.02). Most of the ultrasound studies on the evaluation of sarcopenia have focused on quadriceps femoris [29]. Few studies have focused on the importance of assessing biceps brachii muscle thickness using ultrasound. Abe et al. [45] showed that the muscle thickness of the forearm of the upper limb was significantly correlated to the grip strength. Li et al. [46] showed a positive association between cross-sectional area of the biceps brachii and both ASMI and grip strength in the older adults.

Finally, we found that an increase in RF-VI quadriceps thickness was associated with better mobility on level surfaces while reduced echogenicity associated with improved ability to climb stairs. A recent study evaluates the relationship between the regional and total muscle mass, muscle strength and physical performance comparing older and younger subjects [38]. The authors found that gait speed, grip strength, and regional muscle measurements decreased with age at higher rates (26–28%) than skeletal muscle mass index (15%). Furthermore, gait speed was associated with rectus femoris muscle thickness, but not with skeletal muscle mass index. Consequently, the regional muscle thickness seems to be more important for walking performance than total muscle mass measurements. The lack of early diagnosis of this regional sarcopenia could be important in population groups, such as hip fracture.

The limitations of our study are those of observational studies. The design of the present study is not intended to compare sarcopenia with the risk of malnutrition. There are some inherent risks in using parallel quantitative data to estimate sarcopenia with qualitative data to estimate malnutrition. In addition, working with the elderly population with a hip fracture complicates the inference of results to the rest of the population. Additionally, ultrasound muscle measurement sometimes is difficult in presence of excessive subcutaneous tissue or in absence of patient collaboration. Another limitation could be that in our study the ultrasound examination was performed by a single person. We cannot rule out greater variability if the ultrasound were performed by different operators. However, a recent systematic review showed that ultrasound is a reliable and valid tool for assessing muscle size in older adults [47].

Additionally, processing of the image to calculate muscle echogenicity could depend on the area selected. Finally, we have argued in favor of regional sarcopenia, but this phenomenon is not well described in the literature and there is no consensus regarding that issue. Furthermore, our data base is not sufficient to address that issue in detail.

On the other hand, the strengths of our study were the enhancement of ultrasonography as a valuable tool for or the diagnosis of malnutrition by GLIM criteria and sarcopenia in muscle ultrasound in hospitalized patients for hip fracture. In addition, we consider this pioneering study is able to relate to functional independence. Furthermore, we have described the relationship observed between muscle mass measured by ultrasound and the specific functional capacity of these muscle groups.

## 5. Conclusions

In summary, the thickness of the different muscle groups assessed by ultrasonography shows a correlation with each other. This technique can be complementary to a tool of BIA for assessing patient’s muscle mass and nutritional status. On the other hand, ultrasound measures could detect that the thickness of the masseter is associated with the risk of dysphagia, that of the biceps with the ability to self-feed and mobility and that of the quadriceps RF-VI with mobility in level surfaces and stairs.

## Figures and Tables

**Table 1:** Characteristics of the population according to the studied variables and sex.

Parameter	Total (*n*: 101)	Female (*n*: 71)	Male (*n*: 30)	*P*
Age (years)	86 (9)	86 (10)	86.5 (5)	0.9
Masseter thickness (mm)	10.4 (2.73)	10.35 (2.05)	11.5 (3.2)	0.3
Masseter echogenicity	32.4 (11.7)	35.6 (10.5)	24.5 (11.2)	0.001
Biceps thickness (mm)	22.3 (7.2)	22.2 (7.07)	23.5 (8.4)	0.1
Biceps echogenicity	61.8 (12.2)	62.8 (12.6)	59.3 (11.3)	0.3
Quadriceps thickness (mm)	22.1 (6.45)	22.1 (6.3)	22.2 (6.98)	0.6
Quadriceps echogenicity	63.7 (12.4)	64.8 (11.9)	61 (13.5)	0.3
Xc (ohm)	45.6 (12.2)	45.3 (12.4)	46.4 (11.9)	0.7
Rz (ohm)	509.1 (67.2)	509.1 (65.4)	509.5 (72.8)	0.9
PA (grades)	5.1 (2.3)	5.1 (1.3)	5.1 (1.2)	0.9
ASMI (Kg/m^2^)	6.7 (0.86)	6.6 (0.88)	6.9 (0.75)	0.1
ASMI low (%)	31.7	19.7	60	0.0001
Hand grip (Kg)	13.05 (7.53)	11.9 (6.5)	14.6 (11.65)	0.07
Sarcopenia probable (%) (Hand grip low)	89.1	84.5	100	0.028
Sarcopenia confirmed (%) (Hand grip low + ASMI low)	29.7	16.9	60	0.0001
BMI (Kg/m^2^)	25.39 (4.67)	24.78 (5.69)	26.04 (2.87)	0.09
Arm circumference (cm)	26 (5.9)	26 (6.3)	26.5 (5.5)	0.8
Calf circumference (cm)	31 (4)	31 (4)	31 (5.6)	0.4
Malnutrition risk by MNA-SF (%)	66.3	73.2	50	0.025
Malnutrition by GLIM (%)	43.6	43.7	43.3	0.5
Charlson index	7 (3)	6 (3)	7 (2)	0.1
Barthel index	85 (24)	85 (23)	85 (40)	0.7
Triturated diet (%)	26.8	27.5	25	0.5
Dysphagia (>3 score EAT-10) (%)	13.9	14.1	13.3	0.6
Dysphagia (>15 score EAT-0) (%)	9.9	8.5	13.3	0.3
Feed themselves (%)	71.7	75.6	63.2	0.2
Mobility on level surfaces (%)	60	61	57.9	0.5
Climb stairs (%)	31.7	26.8	42.1	0.2

Xc: impedance, Rz: reactance, PA: phase angle. ASMI: appendicular skeletal muscle mass index; MNA: Mini Nutritional Assessment; BMI: body mass index. P: *p*-value associated to the Mann–Whitney and Chi Square tests for the comparison between females and males.

## Data Availability

Data supporting reported results can be found at Department of Endocrinology and Nutrition, Miguel Servet Hospital, 50009 Zaragoza, Spain; asanzp@salud.aragon.es.
